# Assessing the effectiveness of Enhanced Psychological Care for patients with depressive symptoms attending cardiac rehabilitation compared with treatment as usual (CADENCE): a pilot cluster randomised controlled trial

**DOI:** 10.1186/s13063-018-2576-9

**Published:** 2018-04-02

**Authors:** Suzanne H. Richards, Chris Dickens, Rob Anderson, David A. Richards, Rod S. Taylor, Obioha C. Ukoumunne, Katrina M. Turner, Manish Gandhi, Willem Kuyken, Andrew Gibson, Antoinette Davey, Fiona Warren, Rachel Winder, John Campbell

**Affiliations:** 1 University of Exeter Medical School, Exeter, St Luke’s Campus, Exeter, EX1 2LU UK; 2 University of Leeds Institute of Health Sciences, University of Leeds, Level 10 Worsley Building, Leeds, LS2 9JN UK; 3 NIHR Collaboration for Leadership in Applied Health Research and Care for the South West Peninsula, University of Exeter Medical School, Exeter, St Luke’s Campus, Exeter, EX1 2LU UK; 4 Population Health Sciences, University of Bristol, Canynge Hall, Whatley Road, Bristol, BS8 2PS UK; 5 The National Institute for Health Research Collaboration for Leadership in Applied Health Research and Care West (NIHR CLAHRC West), University Hospitals Bristol NHS Foundation Trust, Bristol, UK; 6 Royal Devon and Exeter NHS Foundation Trust, Barrack Road, Exeter, EX2 5DW UK; 7 University Department of Psychiatry, University of Oxford, Warneford Hospital, 0X3 7JX, Oxford, UK; 8 Health and Social Sciences, University of the West of England, Frenchay Campus, Coldharbour Lane, Bristol, BS16 1QY UK

**Keywords:** Depression, Coronary heart disease, Multimorbidity, Behavioural activation, Mental health care coordination, Cardiac rehabilitation, Randomised controlled trial, Qualitative interviews

## Abstract

**Background:**

Around 17% of people attending UK cardiac rehabilitation programmes have depression. Optimising psychological wellbeing is a rehabilitation goal, but provision of psychological care is limited. We developed and piloted an Enhanced Psychological Care (EPC) intervention embedded within cardiac rehabilitation, aiming to test key areas of uncertainty to inform the design of a definitive randomised controlled trial (RCT) and economic evaluation.

**Methods:**

An external pilot randomised controlled trial (RCT) randomised eight cardiac rehabilitation teams (clusters) to either usual care of cardiac rehabilitation provision (UC), or EPC in addition to UC. EPC comprised mental health care coordination and patient-led behavioural activation with nurse support. Adults eligible for cardiac rehabilitation following an acute coronary syndrome and identified with new-onset depressive symptoms during an initial nurse assessment were eligible. Measures were performed at baseline and 5- and 8-month follow-ups and compared between EPC and UC. Team and participant recruitment and retention rates, and participant outcomes (clinical events, depression, anxiety, health-related quality of life, patient experiences, and resource use) were assessed.

**Results:**

Eight out of twenty teams were recruited and randomised**.** Of 614 patients screened, 55 were eligible and 29 took part (5%, 95% CI 3 to 7% of those screened), with 15 patient participants cluster randomised to EPC and 14 to UC. Nurse records revealed that 8/15 participants received the maximum number of EPC sessions offered; and 4/15 received no sessions. Seven out of fifteen EPC participants were referred to another NHS psychological service compared to none in UC. We followed up 27/29 participants at 5 months and 17/21 at 8 months. The mean difference (EPC minus UC) in depressive symptoms (Beck Depression Inventory) at follow-up (adjusting for baseline score) was 1.7 (95% CI − 3.8 to 7.3; *N* = 26) at 5 months and 4.4 (95% CI − 1.4 to 10.2; *N* = 17) at 8 months.

**Discussion:**

While valued by patients and nurses, organisational and workload constraints are significant barriers to EPC implementation. There remains a need to develop and test new models of psychological care within cardiac rehabilitation. Our study offers important data to inform the design of future trials of similar interventions.

**Trial registration:**

ISRCTN34701576. Registered on 29 May 2014.

Funding details: UK NIHR HTA Programme (project 12/189/09).

**Electronic supplementary material:**

The online version of this article (10.1186/s13063-018-2576-9) contains supplementary material, which is available to authorized users.

## Background

Depression is common in people with coronary heart disease (CHD), affecting approximately a fifth of individuals following an acute coronary syndrome (ACS) [1], coronary artery bypass grafting (CABG) [2] and chronic heart failure [3]. Such depression is important as it is associated with worse health-related quality of life [4–6], greater use of unscheduled care [7, 8], increased health care costs [9] and a doubling of risk of subsequent morbidity and mortality [10–14]. The detection and appropriate management of depression among people with CHD is a policy priority in the UK and in many other countries with well-developed health care systems [15–18].

For most people in the UK who have experienced an ACS or have had coronary revascularisation, cardiac rehabilitation is offered by the National Health Service (NHS) as part of routine care to help people return to optimal functioning. This rehabilitation usually incorporates education, exercise and psychological support, although the exact form of the psychological support provided is not well defined in guidance documents [19] and remains open to interpretation by individual services. While psychological treatments, such as cognitive behavioural therapy, for example, are effective for depression in people with CHD [20, 21], very few rehabilitation services provide access specific psychological treatments part of cardiac rehabilitation. Furthermore, only a minority of rehabilitation services (18% in 2014) provide direct access to specialist psychological care [1]. Thus, despite the availability of evidence-based interventions in primary and secondary care, the majority of people attending cardiac rehabilitation in the UK do not receive adequate treatment for depression.

To improve access to evidence-based psychological treatment for depression among people attending cardiac rehabilitation, we developed and conducted a preliminary evaluation of a complex intervention (Enhanced Psychological Care or ‘EPC’) for delivery by cardiac rehabilitation nurses alongside routine cardiac rehabilitation. Our aim was to pilot the methods and procedures required to undertake a fully powered evaluation of the clinical effectiveness and cost-effectiveness of implementing EPC for patients with new-onset depressive symptoms using cardiac rehabilitation compared with treatment as usual [22]. This paper reports on three specific objectives: to document the flow of patients through a pilot trial (i.e. study eligibility, recruitment and attrition rates); collect participant outcome data to support sample size calculations for a definitive trial; and to establish the optimal data collection methods required to support an economic evaluation. We also undertook qualitative interviews with patients and nurses recruited into the study to explore their views on the acceptability of EPC and of our study procedures. The methods and results of these interviews have been reported elsewhere [23].

## Methods

The full methods employed in the CADENCE study are described in a protocol paper [22].

### Study design

An external pilot cluster randomised controlled trial (RCT) [24, 25], including the piloting of economic data collection, was undertaken. We sought to recruit and randomise eight teams (clusters) delivering a comprehensive cardiac rehabilitation programme to either the intervention (EPC) or control (usual care (UC)) arms in a 5:3 ratio. This randomisation ratio was used to ensure that sufficient nurses and patients were exposed to EPC to support the aims of the qualitative interview study.

Randomisation was carried out by the trial statistician (FW) who was independent of the recruitment of rehabilitation teams and patient participants. The allocation sequence was determined using computer-generated random numbers. Cluster randomisation was balanced by team type (community, hospital or mixed community and hospital teams) and patient throughput at the initial cardiac rehabilitation assessment (‘low’ ≤ 22 patients per month vs ‘high’ > 22 patients per month), the latter facilitating division into small and large teams. The cluster randomised design was essential to avoid contamination between trial arms (for example, it was deemed unrealistic that nurses trained in mental health care coordination would be expected to offer this management approach only to intervention group participants and not those in the control arm). To conceal the allocation sequence from cardiac teams, the cluster randomisation took place immediately after the last team was recruited.

### Interventions tested

#### Usual care (controls)

Usual care was defined as cardiac rehabilitation, commencing at the point the patient attended a specialist cardiac rehabilitation nurse assessment clinic prior to starting a structured rehabilitation programme. The UK British Association of Cardiovascular Prevention and Rehabilitation (BACPR) publish core standards regarding the content of structured rehabilitation, which is commonly delivered in one or two sessions per week for approximately 8 weeks. Sessions last around 2 h and include structured exercise and education (e.g. managing cardiac risk factors) and psychological input (e.g. stress management and/or relaxation training). The 2012 BACPR standards [26] were in effect during this trial (new standards came into effect in 2017 [27]). The 2012 standards advocated the provision of psychosocial care and routine monitoring of mood (anxiety and depression) upon referral and discharge. However, detailed guidance on how psychosocial care should be delivered is not provided. National audit data reports that psychological expertise within cardiac rehabilitation teams is uncommon (< 15%) [1]. Informal discussions with mental health specialists and cardiac rehabilitation staff suggest that there is considerable variation in local cardiac team protocols across the UK, with specialist referrals to mental health services frequently resulting from clinical observation and intuition.

#### EPC (treatment)

A detailed description of the development of our EPC intervention [28], and of the rationale and content supporting its design is reported elsewhere [29]. Briefly, EPC was designed to be embedded within the existing cardiac rehabilitation care pathway and delivered by cardiac nurse specialists supported by an intervention manual. EPC consisted of mental health care coordination [30, 31], including an embedded participant-led behavioural activation (BA) programme [32–37] designed to tackle depressive symptoms. Nurses were trained to apply best-practice clinical decision-making rules [30, 31], matching the intensity of treatment with participant preferences for mental health care. Nurses were also supplied with a manual to support intervention delivery.

The nurse explained the evidence-based treatment options available to all potentially eligible patients. This included BA self-help materials supported by the nurse as part of the cardiac rehabilitation programme, and/or referrals to their general practitioner (GP), local mental health services, or referral to specific cardiac patient psychological support services where available. Nurses were trained to coordinate care while the participant attended cardiac rehabilitation by monitoring depressive symptoms, assessing risk to self or others and agreeing/reviewing any changes to the mental health care plan with participants, including onward referral to other services.

All participants were also offered nurse-supported self-help using a participant BA handbook. The handbook consisted of a structured programme designed to support participants in re-engaging with sources of positive reinforcement from their environment and to develop future strategies for managing their depressive symptoms. A functional analytical approach was adopted, with the handbook designed to help participants develop an understanding of behaviours that interfere with meaningful, goal-oriented behaviours. By teaching the participant to self-monitor mood, individuals were shown how to identify patterns of behaviour associated with their depression. Participants were then encouraged to develop alternative behaviours which were goal-orientated, targeting routine, pleasurable and necessary activities and using diaries to plan these behaviours into daily schedules.

Although our pilot intervention involved a participant-led, self-help BA handbook, nurses were trained to actively support participants. A 2-day training course was delivered by experienced mental health practitioners, during which nurses were trained in key techniques of mental health care coordination, managing safety and risks, and BA. Supported by an intervention manual, nurses were also provided with a session by session guide, detailing the types of coordination actives required as participants moved through their 6–8-week cardiac rehabilitation programme (Additional file 1: Table S1). This guide was flexible to accommodate participant preferences for care. Nurses delivering EPC to participants also received bi-weekly clinical supervision by an experienced mental health practitioner over the telephone.

On completion of their cardiac rehabilitation programme, nurses were required to send structured details of the mental health care that they had delivered to the participant to the participant’s GP. All participants, including those receiving BA and whose depressive symptoms do not respond, were given an opportunity to review their management options with the nurse. Here, treatment response was defined as achieving a minimally clinical important difference (MCID) for the Patient Health Questionnaire-9 (PHQ-9) equivalent to a 5-point reduction in score [38]. It was expected that some people who achieved this MCID remained above the PHQ-9 diagnostic threshold (≥ 10) for depression; as part of their care coordination, these individuals were referred for continuing mental health management upon discharge from cardiac rehabilitation services.

### Settings and study population

We aimed to recruit and randomise eight cardiac rehabilitation teams. We wrote to the lead nurse in each of the 20 operationally distinct teams in South West England, providing detailed information about the study and inviting participation. Each team expressing an interest received a briefing visit, where the trial design and methods were explained by a researcher, and the staff were given an opportunity to ask questions before being asked to provide written consent to participate. All teams agreeing to participate received training in study procedures. Nurses in teams allocated to the EPC intervention arm received EPC training before participant recruitment began.

#### Participant eligibility criteria

Nurses were trained to apply a structured checklist to ascertain participant eligibility. All adult patients (aged 18 years or over) referred for cardiac rehabilitation based on local clinical referral protocols were screened for eligibility. The sample comprised individuals previously admitted with an acute coronary syndrome (unstable angina, non-ST-elevation myocardial infarction (MI) or ST-elevation MI) and/or following a coronary revascularisation procedure (percutaneous coronary intervention (PCI), CABG), with or without heart failure [1].

Patients were routinely screened for depressive symptoms using the nine-item Patient Health Questionnaire (PHQ-9) [39, 40], with individuals scoring 10 or more deemed potentially eligible for inclusion. Patients were excluded if they reported active treatment for depression (psychological or drug therapy) within the previous 6 months. Individuals were also excluded where there was evidence of alcohol or drug dependency, active suicidal intention or having poorly controlled bipolar disorder or psychosis/psychotic symptoms based on a clinical review. Although no language exclusions were applied, we anticipated that the majority of patients would require sufficiently good English language skills to engage with the manual-based EPC intervention, with NHS translation services employed if needed.

### Patient participant recruitment procedure

Eligible participants were invited to take part by a nurse during their initial clinic appointment (prior to commencing a structured rehabilitation programme), and given a brief study sheet to take away and review. The nurse also gained permission to pass the individual’s contact details on to the research team. A researcher then contacted the patient within 7 days to discuss participation and provide them with a detailed Participant Information Sheet. The researcher arranged a visit to obtain written consent and to undertake the baseline trial assessment before the participant commenced the structured rehabilitation programme. Participants were informed that trial participation was voluntary, and that they could withdraw from the study at any time without reason, and that withdrawing would not affect their ongoing clinical care in any way.

### Sample size

Using UK national audit data (2010–2011), 55,452 patients (for MI, PCI, CABG) engaged in cardiac rehabilitation with one of 280 teams (an average of 200 patients per team per year). Assuming that 17% had depressive symptoms [27], this equated to 35 eligible patients per team each year. Establishing participant eligibility and consent rates were key goals of this pilot study as there was no applicable UK data employing a cluster RCT design. For planning purposes we assumed a participation rate of 50%, aiming to recruit 64 patients through the eight teams in 6 months; this target was subsequently amended to 47 participants on completion of a feasibility study (which preceded the pilot trial), which identified that unless the participant recruitment period was extended beyond 6 months, we would be unlikely to achieve our initial target (full details are described elsewhere [29]). This sample size is sufficient to estimate a follow-up percentage as low as 50% with margin of error ± 15% based on the width of the 95% confidence interval (CI), and as high as 90% with a margin of error of ± 13% based on the lower bound of the 95% CI. We did not estimate the intra-cluster (intra-CR team) correlation coefficients (ICC) for key outcomes as the pilot sample was small and estimates would have been imprecise. Using national audit data available during the planning phases of this research (2010–2011; 6272 patients; 119 cardiac rehabilitation teams), we estimated the ICC for depression measured using the Hospital Anxiety and Depression Scale [41] to be 0.047 (95% CI, 0.034 to 0.062). This ICC estimate and CI were used to inform the sample size calculations for the definitive trial.

### Data collection

**Fig. 1:**
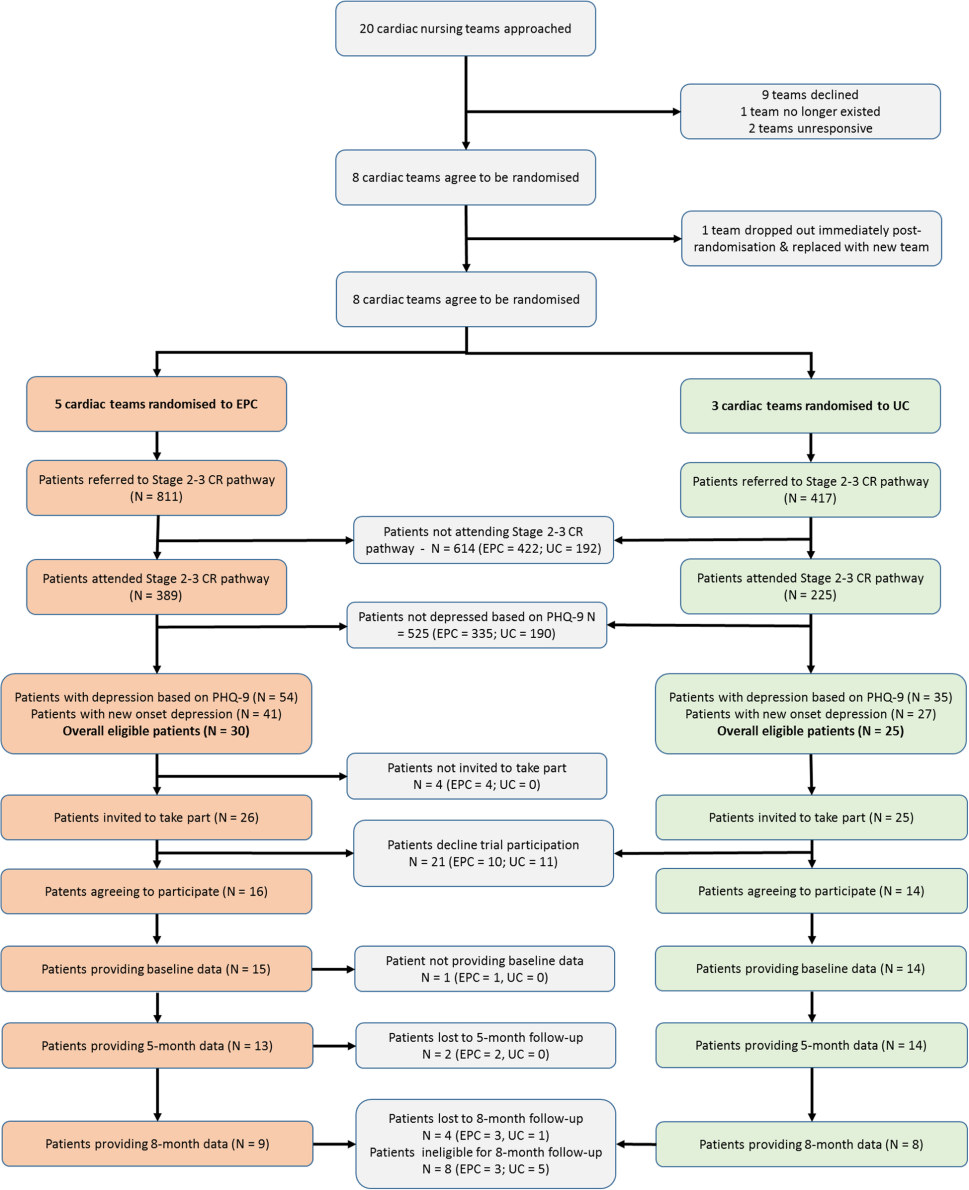
Consolidated Standards of Reporting Trials (CONSORT) Diagram. CONSORT Diagram (with cluster extension) for the CADENCE pilot randomised controlled trial (RCT)

All participants were assessed by a researcher completing measurements on up to three occasions. Baseline measures were completed prior to commencing cardiac rehabilitation, and then two follow-up interviews were scheduled at 5 and 8 months post randomisation (Fig. 1). A summary of the measures collected at each time point can be found in Additional file 1: Table S2. Blinding of patients, practitioners or researchers extracting data on study outcomes was not possible in this cluster design, but the final analysis was carried out by a statistician who was blind to treatment allocation. Data were collected on process measures, patient outcomes, and resource use and costs.

#### Process measures

Process data on study eligibility and participant recruitment were collected by nurses. We extracted data on the number of patients attending an initial nurse assessment, the proportion identified with depressive symptoms during this assessment, the prevalence of new-onset (as opposed to recently treated) depression and the number of patients deemed eligible for trial participation. For eligible patients, we documented the numbers: offered study entry; agreeing to be contacted by a researcher; and those subsequently consenting to take part and undergoing a baseline interview. Participant socio-demographic characteristics (age, sex, Index of Multiple Deprivation (IMD) score [42], ethnicity/preferred language) and underlying cardiac condition were also collected. The proportion of participants completing follow-ups at 5 months (primary endpoint) and 8 months was also reported.

Intervention fidelity was assessed through a review of nurse clinical records. We extracted data on the number of cardiac rehabilitation sessions attended by each participant and the proportion where there was documentary evidence of mental health care coordination in their notes and the type of psychological referrals made (where appropriate). For participants in the EPC arm we also assessed intervention adherence by recording the number of cardiac rehabilitation sessions attended and where the BA component of EPC was reviewed.

#### Participant outcomes measures

##### Baseline assessment only

Although not determining study eligibility, the Revised Clinical Interview Schedule (CIS-R) [43, 44] was administered for the purposes of research to ascertain a clinical diagnosis of depression and other disorders. Participants were also asked for their treatment preferences [45] prior to commencing rehabilitation. Data from clinical records (GP and cardiac nurse notes) were extracted regarding known cardiac risk factors (Body Mass Index (BMI), blood pressure, glycosylated hemoglobin (HbA1c), lipids, smoking status) as recorded at the participant’s latest clinical assessment prior to recruitment.

##### Baseline and follow-up measures

We requested that NHS providers inform us of any participant deaths between baseline and the 8-month follow-up. At the last follow-up we also recorded the date and last recorded known cardiac risk factors in nurse notes. At the same time, we contacted the participant’s GP to arrange for a researcher to extract relevant data from primary care records, including the incidence of any new cardiac events, i.e. death and/or hospital admissions for acute coronary syndromes or revascularisation procedures (CABG or PCI), or mental health events (e.g. self-harm, suicidality) arising since study enrolment. In addition to being outcomes, these were part of the safety monitoring of serious adverse events (SAEs) and adverse events (AE) within the trial.

The baseline and follow-up interviews included participant-reported cardiac-related and non-cardiac-related morbidity and smoking status, antidepressant medication use [46], and resource use. Cardiac nurses used the PHQ-9 to identify and monitor depressive symptoms as part of clinical care. As patients in the EPC arm were likely to complete this tool more often than those in UC, this might differentially impact on its scoring independent of any treatment effects, as EPC participants become familiarised with it. Thus, for the purposes of research, depressive symptom severity was measured using the Beck Depression Inventory (BDI-II) [47, 48] (range 0–63), with minimal (0–13), mild (14–19), moderate (20–28) and severe (29–63) symptom severity ranges specified.

The Beck Anxiety Inventory (BAI) [49, 50] was assessed as anxiety is commonly comorbid with depression. Item scores are summed with total scores of 8–15, 16–25 and 26–63 taken as ranges defining categories for mild, moderate or severe anxiety, respectively. Health-related quality of life (HRQL) was quantified using both generic and disease-specific measures. The EuroQoL Index (EQ-5D-5 L) [51, 52] is a standardised generic HRQL measure commonly used for economic evaluations alongside clinical trials. The HeartQoL questionnaire [53, 54] is a disease-specific measure comprising 14 items covering physical and emotional domains. For both tools lower scores representing poorer HRQL.

To assess patient activation over the course of their EPC, we administered the nine-item Behavioral Activation for Depression Scale – Short Form (BADS-SF) [55], with scores ranging from 0 to 54 and high scores representing greater activation.

Participant experiences of care were assessed at the 5-month follow-up using the self-reported Client Satisfaction Questionnaire (CSQ-8) [56] and an adapted version of the NHS Friends and Family Test (FFT). The CSQ-8 is composed of eight items with scores ranging from 8 to 32 with higher values indicating greater satisfaction. The FFT asks whether you would recommend the care received to family and friends, and includes a single item with a 5-point rating scale (‘Extremely likely’ to ‘Extremely unlikely’) and two free-text items (‘What was good about your experience?’; and ‘What would have made your experience better?’).

### Economic evaluation

We adopted a societal perspective as this is the analytic approach recommended by the UK National Institute for Clinical Excellence. We tested the methods of resource and service-use data collection to enable estimation of the costs to the NHS, costs to social care and personal social services, and relevant costs to patients and their carers/families (including NHS and privately funded mental health care). A preliminary assessment of the cost of providing EPC within cardiac rehabilitation was undertaken. Service-use data were extracted by researchers from routine/administrative sources (e.g. cardiac nurse records, GP records) and participant self-report using the Service and Resource Use Questionnaire (SRUQ). The SRUQ was adapted from the Client Services Receipt Inventory [57], with input from our lay advisors. The completeness and reliability of participant reporting was compared with routine administrative data for the types/amounts of health care/service used over the follow-up periods.

### Statistical analysis plan

As this is a pilot study we do not report definitive estimates of effectiveness and costs. Pilot study data were reported according to the Consolidated Standards of Reporting Trials (CONSORT) extension for cluster randomised trials [58]. Recruitment, intervention and control uptake, outcome completion rates and dropout rates were reported with 95% CIs. Recruitment rates and participant characteristics at baseline were compared between the trial arms to assess whether there was evidence of selection bias resulting from cluster randomisation which took part prior to participant recruitment [59].

Baseline characteristics of the participants were summarised by trial arm status using means and standard deviations (SDs) (or medians and interquartile ranges) for continuous variables, and numbers and percentages for categorical variables. Participant-reported outcomes were compared between trial arms (i.e. EPC minus UC), adjusting for baseline scores, using linear regression for continuous variables and logistic regression for binary variables based on an intention-to-treat analysis. As this is a pilot study we reported only 95% CIs and no *p* values. We did not use analytical methods that allow for clustering because the number of clusters and participants per cluster was too small. For this reason, and the fact that this is a pilot study, the CIs should be interpreted cautiously. We carried out a complete case analysis, including only those participants who provided outcome data. No data were imputed as this is not a definitive trial of intervention effectiveness.

In addition, as part of piloting the BDI-II, treatment effects regarding depression symptom severity were reported in five ways: (1) mean (SD) and between-group mean difference; (2) the proportion of participants demonstrating a reduction in score from baseline by > 50% (deemed a clinically important ‘response’ to treatment [60]); (3) the proportion of participants demonstrating remission (going from scores ≥ 14 to < 14); (4) the proportion of participants demonstrating a minimal clinically important difference (MCID) in the BDI-II scores of 17.5%; [61] and (5) a clinically significant and reliable change (CSRC), where participants meet two criteria: (a) passing below the BDI-II remission threshold (see (3) above) and (b) establishing that the magnitude of the participant’s change in score is statistically reliable, i.e. the difference between the participant’s scores at baseline and follow-up was calibrated by the standard error of the difference between two scores [62]. All statistical analysis was undertaken in Stata v14.

### Ethical and governance considerations

The Royal Devon and Exeter NHS Foundation Trust acted as the trial sponsor. The CADENCE study protocol (version 4, 19 June 2015), was reviewed and received a favourable ethical opinion from the NHS National Research Ethics Service Committee South West – Exeter (reference: 14/SW/0139) and the relevant NHS research governance approvals were obtained. All nurse and patient participants provided written consent prior to participating in this study.

This study is registered with the IRCTN trials registry (ISRCTN34701576) and was adopted by the UK Comprehensive Research Network (UKCRN ID: 17105). All researchers and nurses involved in participant recruitment undertook Good Clinical Practice (GCP) training.

### Patient and public involvement

Patient and public involvement (PPI) was sought during protocol development following a published framework for PPI involvement in research [63]. Four lay advisors with relevant lived experience, reviewed and commented on our research proposal. PPI continued throughout the project, with the precise roles of advisors negotiated across time. In the early stages, our advisors reviewed outcome measures and informed the design of all study-related materials, including Participant Information Sheets and the development of intervention manuals. Lay advisors also attended project management meetings on a quarterly basis and actively participated in decisions related to the ongoing running of the project; for example, advising on the acceptability to patients of modifications to EPC intervention.

## Results

### Recruitment

The recruitment and flow of cardiac teams and patient participants are summarised in Fig. 1. Between December 2014 and February 2015, 20 cardiac rehabilitation teams were approached, of which eight agreed to participate (40%), nine declined, two did not respond and one ceased to exist due to service reconfiguration. Teams were then randomised (five to EPC and three to usual care (UC)), with one team withdrawing from the EPC arm immediately post randomisation. This team was replaced with another team recruited from the West Midlands that was similar with respect to patient throughput and population mix.

Of the eight participating teams, two were hospital based, three were community based and the remainder based both in the community and at the hospital (Table 1). Team size and throughput varied considerably, with teams comprised of between one and seven cardiac nurses working either part-time or full-time, and providing care for between 12 and 70 patients per month. Most of the teams offered centre-based programmes either in the community and/or at the hospital, clinic-based individual appointments and home-based programmes with one team offering only centre-based programmes. The majority of teams were in urban and rural settings, with only two teams serving inner-city patient populations. The three UC teams had no access to psychological support integrated within their cardiac rehabilitation programme, although one team reported having limited access to a clinical psychologist. Three of the EPC teams had little or no access to psychological support; the other two EPC teams, located in inner city areas, had established close links to the clinical psychology and community mental health services.

**Table 1:** Characteristics of participating sites at baseline

Team	Allocation	Team location (setting)	Cardiac team clinical staffing	Type of cardiac rehabilitation programme offered (including usual psychological care^a^)	*N* patients assessed per month^b^
A	UC	Hospital (rural)	7 nurses (2 full-time; 5 part-time)	Centre-based groups, Clinic-based individual appointments, Home-based programmes	40
B	UC	Community (rural)	7 nurses (1 full-time; 6 part-time)	Centre-based groups – with some support from a clinical psychologist, Clinic-based individual appointments, Home-based programmes	60
C	UC	Community (rural)	3 nurses (part-time), 1 team manager (part-time)	Centre-based groups	12–16
D	EPC	Community (rural/urban)	2 nurses (1 full-time; 1 part-time)	Centre-based groups, Clinic-based individual appointments, Home-based programmes	26
E	EPC	Hospital (rural/urban)	2 nurses (part-time)	Centre-based groups, Clinic-based individual appointments, Home-based programmes	70
F	EPC	Mixed hospital + community (urban)	1 nurse (part-time)	Centre-based groups, Clinic-based individual appointments, Home-based programmes	32
G	EPC	Mixed hospital + community (inner-city)	4 nurses (part-time)	Centre-based groups – limited psychological input via referral to community mental health workers, Clinic-based individual appointments, Home-based programmes	33
H	EPC	Mixed hospital + community (inner-city)	6 nurses (part-time)	Centre-based groups, Clinic-based individual appointments – part-time clinical psychologist linked to team, Home-based programmes	50–60

*EPC* Enhanced Psychological Care, *UC* usual care

^a^Unless stated there is no dedicated psychological support available (either within the team, or through an established referral pathway)

^b^Data derived from the team profile questionnaire completed prior to randomisation. Teams reported the ‘average’ number of patients assessed per month

Due to unforeseen delays in securing regulatory approvals in some areas, individual teams recruited patients for between 3 and 6 months during the period June 2015 to December 2015. A total of 614 patients (389 EPC arm, 225 UC arm) were screened for eligibility, of whom the mean age was 66.2 years (range 32 to 95 years), 70.2% were male (481/614) and 94.3% reported their ethnicity as ‘White’ (579/614). Eighty-nine out of 614 (14.5%) scored 10 or more on the PHQ-9, of whom 55/614 were deemed eligible for the trial (9%, 95% CI 7% to 12%); 33 were ineligible as they were either actively being treated for depression (*n* = 21, 3% 95 CI 2% to 5%), or excluded for other reasons (e.g. alcohol and/or drug dependency problems, were currently ineligible for cardiac rehabilitation based on their cardiac condition, or had language and communication problems).

Fifty-one of the 55 patients deemed to be eligible by a nurse were invited to participate. Of the four patients not invited, one patient declined any further input from the cardiac rehabilitation service, two patients were not offered EPC as the nurse was at capacity and could not take on more patients, and the final patient reported a marked improvement in mood and did not feel that they required EPC. Of the 51 patients offered study entry, 21 declined participation (41%) for a variety of reasons including: a preference to see their GP (*n* = 6); feeling better and would prefer to see how mood goes while taking part in cardiac rehabilitation (*n* = 3); that their mood was a long-standing problem unrelated to this current hospital stay (*n* = 2); or they did not want the EPC intervention (*n* = 2) (nine patients did not give a reason). Thus, of the 55 eligible patients, 30 (54%, 95% CI 41 to 68%) were initially recruited (one of whom withdrew prior to providing baseline data) and 29 participants were effectively (cluster) randomised (15 EPC; 14 UC), equivalent to 5% (95% CI 3 to 7%) of the 614 patients screened.

At the 5-month follow-up 27/29 participants completed an assessment (93%, 95% CI 77 to 99%). At 8 months we were unable to collect data for 8/29 participants, due to slower than anticipated recruitment to the trial such that these participants could only contribute data to the 5-month follow-up before data collection closed due to funding constraints. Of the 21 participants for whom 8-month data could have been obtained, 17 (81%, 95% CI 58 to 95%) provided data.

### Sample characteristics

Of the 51 eligible participants who were offered study entry, 15/29 (52%) male patients and 15/22 (68%) female patients accepted. Of 49 eligible White patients invited to participate, 19 declined (39%), as did both of the two Asian/Asian British patients. The mean age among 30 eligible patients who accepted participation was 63.8 years (SD 9.5), range 47 to 78 years; among 21 patients who declined, mean age was 61.1 years (SD 12.3), range 36 to 83 years. For the acceptors, mean PHQ-9 score was 13.4 (SD 4.1), range 10 to 25; for the decliners, mean PHQ-9 score was 14.4 (SD 4.6), range 10 to 24.

The socio-demographic characteristics, smoking status, and health status of trial participants are reported in Table 2. While both trial arms were broadly similar across a range of baseline characteristics, there was some imbalance, with the EPC arm having a greater proportion of participants in the three most deprived deciles compared with UC (40% (6/15) versus 14% (2/14)). Despite recruiting in some ethnically diverse areas, all participants reported their ethnicity to be White. Some imbalance was also observed regarding baseline health characteristics. The EPC group reported higher levels of depression at baseline compared with UC (mean PHQ-9 score 14.2 versus 11.9) and BDI-II (18.4 versus 12.5); 67% (10/15) participants in EPC had a BDI-II score of ≥ 14 compared with 50% (7/14) in UC.

**Table 2:** Participant characteristics at baseline

	UC, *N* = 14	EPC, *N* = 15
Gender; *n* (%)		
Male	7 (50)	8 (53)
Age (years); mean (SD)	68.1 (8.6)	62.7 (8.9)
Ethnicity; *n* (%)		
White	14 (100)	15 (100)
Preferred language; *n* (%)		
English	13 (93)	15 (100)
Other	1 (7)	0 (0)
Smoking status; *n* (%)		
Never smoked	5 (36)	4 (27)
Ex-smoker	9 (64)	11 (73)
Time since quitting smoking (ex-smokers only); *n* (%)		
Less than 6 months	3 (33)	3 (27)
6 to 12 months	0 (0)	0 (0)
1 to 5 years	0 (0)	2 (18)
5 to 10 years	1 (11)	0 (0)
More than 10 years	5 (56)	6 (55)
Index of Multiple Deprivation median decile (IQR) (lower deciles are more deprived)	6 (4, 8)	5 (1, 7)
Index cardiac event and/or and procedures^a^		
ACS and/or revascularisation	11 (79)	11 (73)
Other heart condition^b^	3 (21)	1 (7)
Other cardiac procedure (pacemaker, valve surgery)	0 (0)	3 (20)
CIS-R primary diagnosis category; *n* (%)	*N* = 13^c^	*N* = 14^c^
No psychiatric diagnosis identified	9 (69)	6 (43)
Mixed anxiety and depressive disorder – mild	1 (8)	1 (7)
Mild depressive episode	3 (23)	4 (29)
Moderate depressive episode	0 (0)	3 (21)
Secondary diagnosis category; *n* (%)		
No psychiatric diagnosis identified	12 (92)	9 (64)
Mixed anxiety and depressive disorder – mild	1 (8)	1 (7)
Generalised anxiety disorder – mild	0 (0)	1 (7)
Mixed anxiety and depressive disorder	0 (0)	2 (14)
Specific (isolated) phobia	0 (0)	1 (7)
Emotional health		
PHQ-9; mean (SD)	11.9 (1.8)	14.2 (4.7)
BDI; mean (SD)	12.5 (4.9)	18.4 (7.4)
BAI; mean (SD)	12.5 (4.4)	19.3 (10.7)
Do you believe you have low mood?; *n* (%)		
Yes	11 (79)	11 (73)
No	3 (21)	4 (27)
Do you want any professional help for your low mood?; *n* (%)^d^		
	*N* = 11	*N* = 11
Strongly prefer help	1 (9)	5 (45)
Prefer help	4 (36)	2 (18)
Prefer not to receive help	3 (27)	3 (27)
Strongly prefer not to receive help	3 (27)	1 (9)
What type of professional help would you prefer?; *n* (%)^d^		
	*N* = 11	*N* = 11
Strongly prefer non-drug help	6 (55)	9 (82)
Prefer non-drug help	3 (27)	0 (0)
Prefer drug-based help	1 (9)	0 (0)
Strongly prefer drug-based help	0 (0)	0 (0)
Do not mind	1 (9)	2 (18)
Health-related quality of life		
EQ-5D-5 L; mean (SD)	0.801 (0.097)	0.644 (0.226)
EQ-5D VAS; mean (SD)	63.0 (23.5)	48.7 (19.3)
HeartQoL; mean (SD)	21.3 (9.1)	15.2 (9.9)

*ACS* acute coronary syndrome, *BAI* Beck Anxiety Inventory, *BDI-II* Beck Depression Inventory, *CIS-R* Revised Clinical Interview Schedule, *EPC* Enhanced Psychological Care, *EQ-5D-5 L* EuroQoL Index, *IQR* interquartile range, *PHQ* Patient Health Questionnaire-9, *SD* standard deviation, *UC* usual care, *VAS* Visual Analogue Scale

^a^Clear diagnostic information was not always present

^b^Includes atrial fibrillation, chest pain, heart failure or ischaemic heart disease

^c^Some participants did not complete this component of the baseline assessment

^d^Only completed by participants who believed themselves to have low mood

The precipitating cardiac events as recorded in GP or cardiac nurse records at baseline are reported in Table 2. The majority of patients attended cardiac rehabilitation following an acute coronary syndrome and/or revascularisation, and these were equally distributed between the two groups.

### EPC delivery

Seven nurses from four teams provided EPC to one or more participants (one team recruited no participants to the study). Three teams had two nurses providing EPC and one team had a single practitioner. Most nurses had their own participants allocated to them, occasionally sharing cases when the allocated nurse was unavailable. Thirty clinical supervision calls were made to the teams over a 7-month period.

The number of cardiac rehabilitation sessions attended recorded in nurse notes ranged from 0 to 22 (mean 8.3, SD 8.1) for EPC participants and 1 to 12 (mean 7.4, SD 2.7) in UC (Table 3). Consistent with the training provided, there was documentary evidence that a maximum of eight sessions of EPC were offered. Approximately half received the maximum number of sessions (8/15, 53%), while 4/15 (27%) of participants had no EPC sessions recorded (Table 3). Documentary evidence of mental health care coordination activities was observed for 47% (7/15) of EPC participants referred on to another NHS service (e.g. GP or another psychological care service). This compared with 0/14 (0%) of UC participants. No participants in either arm self-reported accessing private mental health care.

**Table 3:** Process data for delivery of Enhanced Psychological Care (EPC) at 8-month follow-up

Outcome	Usual care (UC), *N* = 14	EPC, *N* = 15
Number of cardiac rehabilitation sessions; median (min, max)		
Offered	8 (2, 14)	7 (0, 24)
Attended	7.5 (1, 12)	7 (0, 22)
Number of EPC sessions attended; *n* (%)		
8	N/A	8 (53)
7		1^a^ (7)
6		1^b^ (7)
1		1^c^ (7)
0		4 (27)
Days elapsed from consent date to starting cardiac rehabilitation; median (min, max)	3 (− 15, 27)	5 (− 35, 22)
Participants who started cardiac rehabilitation prior to trial recruitment; n/*N* (%)	6 (43)	2 (13)
Days elapsed from trial entry to start of EPC; median (min, max)	N/A	5 (0, 19)
Days elapsed from trial entry to discharge from cardiac rehabilitation; median (min, max)	69 (13, 112)	89 (48, 185)
Evidence of psychological care coordination activities provided in nurse notes; *n* (%)	N/A	5 (33)
Referral to GP/other psychological care services; *n* (%)	0 (0)	7 (47)^d^

^a^Participant had only seven sessions scheduled

^b^Participant had eight sessions scheduled but did not attend two sessions

^c^Participant had only one session scheduled

^d^For some patients, there was no evidence of psychological care coordination in the nurse notes, but there evidence of onward referral describe in the GP discharge letter

### Clinical outcomes

No deaths were recorded in either arm of the trial between baseline and the final follow-up. Eight new cardiac events/diagnoses were recorded between baseline and the 5-month follow-up period and a further four events occurred between 5 months and 8 months (Table 4).

**Table 4:** Cardiac events derived from general practitioner (GP) or cardiac nurse records at follow-up

	Baseline to 5 months		5 months to 8 months	
Cardiac event^a^; *n*	UC, *N* = 14	EPC, *N* = 13	UC, *N* = 8	EPC, *N* = 9
ACS or revascularisation	2	3	0	1
Other diagnosis^b^	2	1	1	2

*ACS* acute coronary syndrome, *EPC* Enhanced Psychological Care, *UC* usual care

^a^Multiple outcomes can occur in an individual participant

^b^Other diagnoses recorded include atrial fibrillation, heart failure, ischaemic coronary disease and left ventricular dysfunction

Between baseline and the 5-month follow-up only two participants (one EPC, one UC) had evidence of a mental health event related to a newly diagnosed condition recorded in GP records, and two participants (one EPC, one UC) had evidence of prescribing antidepressant medication.

To test the feasibility of collecting physiological and biochemical measurements from routine records, we assessed data availability from either GP or cardiac nurse records. The routine recording of such measurements was highly variable (Additional file 1: Table S3); blood pressure measurements were the most frequently reported, with other measures (HbA1c and triglycerides) frequently omitted.

During the trial, the protocol relating to managing self-harm/suicide risk was triggered for three participants. Three of these were recorded by the nurse during an EPC intervention session and one managed by a researcher during an assessment. For each participant, the initial risk was identified as the individual reporting plans/preparations to self-harm but with protective factors identified, and the individual advised to seek help from their GP. One participant was subsequently escalated to a higher risk as they had taken no action to talk to their GP regarding their mood, and their GP was contacted (with the participant’s permission) to alert them to the risk.

Eleven serious adverse events relating to six participants were recorded, including participant admissions to hospital on account of infective conditions, asthma or cardiac complications (e.g. chest pain, additional stent requirement, postural hypotension). None were judged to be related to the trial intervention or research procedures by an independent monitoring committee.

### Participant-reported outcome measures

Participant-reported outcomes reported at 5- and 8-month follow-up are presented in Table 5. In terms of depressive symptoms, the mean difference (EPC minus UC) in BDI-II score at follow-up (with adjustment for baseline score) was 1.7 (95% CI − 3.8 to 7.3; *N* = 26) at 5 months and 4.4 (95% CI − 1.4 to 10.2; *N* = 17) at 8 months. Around half of participants with data available at both baseline and 5 months (*N* = 26) had a 50% reduction in BDI-II at 5 months. A 17% reduction from baseline symptoms (the MCID) was observed for 10/12 (83%) in EPC and 11/14 (79%) in UC, with 43% (3/7) and 86% (6/7), respectively, considered to be in remission (BDI < 14) at 5 months. Among those in remission, in the EPC arm 2/7 (29%) had a clinically significant and reliable change, compared with 3/7 (43%) in UC.

**Table 5:** Participant-reported mental and physical health outcomes at 5-month and 8-month follow-up

Outcome variable	5 months			8 months		
	UC, *N* = 14	EPC, *N* = 13	Mean difference or OR^a^ (95% CI)	UC, *N* = 8	EPC, *N* = 9	Mean difference or OR^a^ (95% CI)
BDI-II; mean (SD), *n*	7.4 (3.7), 14	10.2 (8.4), 12	1.7 (− 3.8, 7.3)	7.0 (3.5), 8	12.6 (6.6), 9	4.4 (− 1.4, 10.2)
50% reduction in BDI-II from baseline; *n*/*N* (%)	7/14 (50)	5/12 (42)	0.71 (0.16, 3.25)	4/8 (50)	2/9 (22)	0.29 (0.04, 2.09)
Remission (< 14 BDI-II) from baseline^b^; *n*/*N* (%)	6/7 (86)	3/7 (43)	0.13 (0, 1.37)	4/4 (100)	2/5 (40)	0 (0, 1.10)
BDI-II MCID (17.5% reduction post baseline); *n*/*N* (%)	11/14 (79)	10/12 (83)	1.36 (0.22, 8.31)	5/8 (63)	6/9 (67)	1.20 (0.18, 7.99)
BDII-II CSRC from baseline^c^; *n*/*N* (%)	3/7 (43)	2/7 (29)	0.53 (0.07, 4.33)	2/4 (50)	1/5 (20)	0.25 (0, 3.56)
BAI; mean (SD), *n*	9.2 (4.0), 14	14.7 (8.3), 13	4.6 (− 0.8, 10.0)	6.4 (4.6), 8	12.7 (7.1), 9	5.0 (− 1.2, 11.1)
EQ-5D-5 L; mean (SD), *n*	0.875 (0.115), 13	0.885 (0.065), 13	0.045 (− 0.023, 0.113)	0.876 (0.092), 8	0.827 (0.116), 9	− 0.041 (− 0.145, 0.064)
EQ-5D VAS, mean (SD), *n*	72 (21), 14	72 (21), 12	5 (− 13, 22)	64 (18), 7	64 (12), 8	1 (− 17, 19)
HeartQoL; mean (SD)	31.5 (4.9), 14	22.9 (10.3), 13	− 8.2 (− 14.9, − 1.4)	28.3 (6.7), 8	28.6 (7.6), 9	0.1 (− 7.9, 8.0)

*BAI* Beck Anxiety Inventory, *BDI-II* Beck Depression Inventory, *CI* confidence interval, *CSRC* clinically significant and reliable change, *EPC* Enhanced Psychological Care, *EQ-5D L* EuroQoL Index, *MCID* minimally clinical important difference, *OR* odds ratio, *SD* standard deviation, *UC* usual care, *VAS* Visual Analogue Scale

^a^Between-group comparison. The mean difference is adjusted for baseline score. No adjustments for clustering were made

^b^At baseline, 7/14 UC participants and 10/15 EPC participants had a BDI-II score ≥ 14; participants with scores ≤ 13 at baseline excluded

^c^Clinically significant and reliable change can only be calculated for participants with a baseline BDI-II score of ≥ 14

In terms of anxiety, the mean difference (EPC minus UC) in the BAI was 4.6 (95% CI − 0.8 to 10.0) at 5-month follow-up and 5.0 (95% CI − 1.2 to 11.1) at the 8-month follow-up.

In terms of HRQL, the mean difference in EQ-5D-5 L index and Visual Analogue Scale (VAS) scores (adjusted for baseline score) between EPC and UC at 5-month follow-up was 0.045 (95% CI − 0.023 to 0.113) and − 0.014 (95% CI − 0.145 to 0.064), respectively. At 8 months the mean differences were − 0.041 (95% CI − 0.145, 0.064) for the EQ-5D-5 L index and 1 (95% CI − 17, 19) for the VAS scores, respectively. The mean difference in HeartQoL (adjusted for baseline score) between EPC and UC at 5-month follow-up was − 8.2 (95% CI − 14.9 to − 1.4), although by 8 months, this difference was negligible (0.1, 95% CI − 7.9 to 8.0).

At 5 months, participants in both arms reported a positive experience of their care on both the CSQ-8 and the FFT (Table 6), although there was some evidence that individuals receiving EPC reported higher satisfaction levels. For example, all the participants in the EPC arm were extremely satisfied with the amount of help they received (11/11), and most of the participants in the UC arm were extremely satisfied (8/13). The majority of participants in both arms were extremely likely to recommend the service to friends and family (EPC = 11/13; UC = 10/14).

**Table 6:** Patient experience ratings from the Client Satisfaction Questionnaire (CSQ) and the Friends and Family Test (FFT) at 5-month follow-up

Patient-reported outcome; *n* (%)	UC, *N* = 14	EPC, *N* = 13
*Client Satisfaction Questionnaire*		
How would you rate the quality of service you have received?	*N* = 13	*N* = 11
Excellent	9 (69)	9 (82)
Good	3 (23)	2 (18)
Fair	1 (8)	0 (0)
Poor	0 (0)	0 (0)
Did you get the kind of service you wanted?^a^	*N* = 13	*N* = 11
Yes, definitely	8 (62)	10 (91)
Yes, generally	5 (38)	1 (9)
To what extent has our programme met your needs?^a^	*N* = 13	*N* = 11
Almost all of my needs have been met	9 (69)	9 (82)
Most of my needs have been met	3 (23)	2 (18)
Only a few of my needs have been met	1 (8)	0 (0)
If a friend were in need of similar help, would you recommend our programme to him or her?^a^	*N* = 14	*N* = 11
Yes, definitely	9 (64)	11 (100)
Yes, I think so	5 (36)	0 (0)
How satisfied are you with the amount of help you have received?^a^	*N* = 13	*N* = 11
Very satisfied	8 (62)	11 (100)
Mostly satisfied	5 (38)	0 (0)
Have the services you received helped you to deal more effectively with your problems?^a^	*N* = 13	*N* = 11
Yes, they helped a great deal	8 (62)	9 (82)
Yes, they helped somewhat	5 (38)	2 (18)
In an overall, general sense, how satisfied are you with the services you have received?^a^	*N* = 13	*N* = 11
Very satisfied	9 (69)	10 (91)
Mostly satisfied	4 (31)	1 (9)
If you were to seek help again, would you come back to our programme?^a^	*N* = 13	*N* = 11
Yes, definitely	7 (54)	10 (91)
Yes, I think so	4 (31)	1 (9)
No, I don’t think so	2 (15)	0 (0)
*Friends and Family Test*		
How likely are you to recommend this help or support to friends and family if they needed similar care or treatment?^a^	*N* = 14	*N* = 13
Extremely likely	10 (71)	11 (85)
Likely	4 (29)	0 (0)
I did not receive any help or support	0 (0)	2 (15)

*EPC* Enhanced Psychological Care, *UC* usual care

^a^Responses were highly skewed to positive assessments. Unless reported, no participants used the neutral or negative response categories provided

### Calculating the required samples size for a future trial

Assuming an ICC of 0.05, we estimated the sample size in each of scenarios for a future, definitive cluster RCT based on the BDI-II MCID observed at the 8-month follow-up Additional file 1: Table S4). An adequately powered definitive cluster RCT would require 50 cardiac teams and 650 participants (13 participants recruited per team), randomising 25 cardiac teams and 325 participants to each trial arm. This sample size is large enough to detect an effect size of 0.35 SD units on the BDI-II with 90% power at the two-sided 5% level of significance. Consistent with our pilot data, this calculation assumed 80% follow-up at participant level at 8 months. Using pilot data, on average it took a cardiac team 1.38 months to recruit a participant. Thus, the length of recruitment for the definitive trial is estimated to be 18 months (13 × 1.38).

### Methods to support economic evaluation

#### Costing the intervention

The estimated elements of costs, and the cost per participant receiving the EPC intervention is presented in Table 7.

**Table 7:** Per patient cost of delivering Enhanced Psychological Care (EPC)

	Units^d^	Unit costs £	Cost £
Initial costs			
For 15 nurses attending training (trainers’ and facilitator’s time, delivery and preparation)	45 + 41 h^a^	Various	7157
Nurse training (nurses’ time)	56 + 21 h^c^	35^b^	2695
Nurse manuals (printing)	8 manuals	4	32
Ongoing costs			
Telephone clinical supervision of behavioural activation (BA) delivery	35 h	50	1750
Nurses’ time for having supervision (estimated 50 h overall)	50 h	35^b^	1750
Total overhead cost of providing EPC			13,384
Number of participants in EPC trial arm			15
Mean allocated overhead cost of EPC per patient (in feasibility trial)			892
Per participant nurse time for delivering EPC within cardiac rehabilitation sessions	1.8 h^c^	35^b^	63
Patient booklet print costs	1	4	4
Estimated cost per EPC recipient			959

^a^Two figures presented as EPC training was delivered to two different nurse cohorts

^b^Cost per working hour for a Band-5 hospital-based nurse (excluding qualification costs). Unit Costs of Health and Social Care 2015, PSSRU

^c^Mean of 7.8 rehabilitation sessions attended. First, mid-point and final session 20 min each; other 4.8 sessions 10 min each = mean of 1 h 48 mins per participant in total

^d^Data sources: trial records (e.g. invoices) or interviews with relevant study personnel, unless otherwise stated

The calculation of intervention costs makes no adjustment for the small scale of the pilot trial, and so it is inevitably an overestimate of what the actual cost would be if the intervention were to be delivered routinely to larger numbers of patients. For example, here the initial nurse training costs account for a significant proportion of the overall cost of providing EPC, and the relative contribution to the cost per patient will reduce as a larger number of patients receiving EPC over a longer period. The estimated time that nurses take to deliver EPC elements within rehabilitation accounts for a relatively small amount of the overall cost (£63 of the estimated £959 per participant).

Participants self-reported service use in the time periods (baseline to 5 months, and 5 months to 8 months). The types of health service used and associated costs are presented in the Additional file 1: Table S5. All participants reported using GP services in both periods, and the majority also had hospital outpatient appointments. Although a minority had hospital inpatient stays, these account for a large proportion of the total costs of health care use.

Due to the small sample size, any differences in costs between the trial arms should not be interpreted, as they could easily be due to chance. However, these costs could provide useful point estimates and SDs for planning future definitive randomised trials of similar interventions in similar patient groups, as do the data presented previously on HRQL and EQ-5D-5 L index scores (Table 5).

#### Sources of data to support economic evaluation

Service-use data for the total 8-month follow-up period were available from GP records for 27/29 participants and via self-report for 17/29 participants. For the 17 participants with data from both sources, we calculated the level of agreement for the number of GPs or practice nurses visits, emergency department visits and inpatient hospital admissions (Additional file 1: Table S6). The lowest agreement was observed for the number of visits to GPs or practice nurses, with no participants recalling the same number of GP visits as recorded in GP notes, and only 3/17 (18%) of participants recalling the same number of practice nurse visits. In contrast, 11/17 (65%) participants recalled the same number of emergency department attendances as their GP records show, and 14/17 (82%) of participants recalled the same number of hospital inpatient stays. While the crude agreement was variable for all four types of health service use there seems to be no systematic tendency to either over-estimate or under-estimate service usage. For example, while crude agreement between sources was poor for GP visits, the mean number of GP visits was the same whether calculated from self-report or GP records data.

Cardiac nurse notes only consistently recorded the use of hospital services and thus the level of agreement could only be estimated for the number of hospital admissions reported by participants and nurse records. The crude agreement was high (15/17; 88%) between participant reports and nurse records of inpatient stays for the 8-month follow-up period. The mean (SD) number of self-reported hospital admissions was 0.5 (0.9) compared with 0.3 (0.6) as recorded in nurse notes (intraclass correlation coefficient = 0.55). No participants underestimated the number of hospital stays, although two individuals overestimated hospital admissions.

## Discussion

In this pilot trial, we successfully tested the methods and procedures required to undertake a fully powered evaluation of using cardiac rehabilitation nurses to implement EPC for their patients with new-onset depression. We recruited eight cardiac rehabilitation nursing teams drawn from a range of settings in South West England and the West Midlands, and compared a range of outcomes against those observed in UC. While the cluster randomisation process was successfully implemented, the withdrawal of one randomised team required responsive action from the research team.

Of 614 patients screened for eligibility over a 6-month recruitment period, 55 met trial eligibility criteria, of whom 29 provided baseline data. Follow-up rates at 5- and 8- months post baseline were 93% and 81%, respectively. A further 21 patients met baseline eligibility criteria, but were not eligible for inclusion on account of having received pre-existing treatment for depression. We were unable to recruit patients from ethnic minority backgrounds. This, and other observed imbalances between the two trial arms, are likely to be addressed in a larger, fully powered study.

We successfully collected participant outcome data to inform key design parameters of a future, definitive trial, such as recruitment and attrition rates, and participant-reported outcomes. In addition, we undertook a case note review of cardiac nurse and GP notes. A review of these procedures suggested that a future trial might require capture of data from case note review of cardiology records for some domains. Collection of BDI-II data proved satisfactory with only minimal missing data. Although the number of patients completing the 8-month follow-up was small, we were able to estimate the distribution of BDI-II scores, and investigate several approaches to calculate treatment effects for depression status at follow-up. We were also able to show that an adequately powered definitive cluster RCT would be challenging. The National Audit of Cardiac Rehabilitation identified 266 cardiac rehabilitation teams in England in 2013–2014 [1]. Our sample size calculations estimated that a definitive RCT would be required to recruit 50 cardiac teams and 650 participants (recruiting for 18 months) to detect a clinically meaningful difference in depression severity (BDI-II) between EPC and UC arms.

In undertaking our economic evaluation, relevant data were captured with a view to identifying costs across the whole system of care. Service-use data were collected from routine and administrative sources, as well as via participant self-report. We encountered challenges in identifying time spent by nurses in delivering EPC since this was distributed across all of the nurse interactions with the patient rather than in discrete, quantifiable periods of consultation time. Our study found some evidence to support the use of self-report service-use data, particularly regarding recall of emergency department attendance, hospital admission and GP attendance. Although intensive, requiring considerable researcher effort, our data capture processes appeared satisfactory.

The findings from a nested qualitative study have been reported elsewhere [29], the core findings of which are summarised here. Some patients who might have benefitted from EPC did not attend subsequent cardiac rehabilitation sessions. In addition, some eligible patients were not offered EPC because of nurse workload. Some patients chose not to take part in the trial, reporting that they did not identify themselves as experiencing ‘low mood’, or did not wish to receive more information relating to their cardiac event. EPC participants, however, viewed one-to-one dedicated nurse time addressing psychological issues as important in helping them achieve timely recovery from their depression. In addition, some patients identified and valued the holistic approach to care. Despite this, it was clear that explanation of BA as a key component of EPC was needed, and that approaches to delivering the intervention need to be tailored to the needs of the individual.

Qualitative interviews with nurses [29] revealed that they felt equipped to deliver the EPC intervention, and valued training in mental health care coordination and mental health risk assessment, particularly in respect of the use of the PHQ-9 instrument during their initial screening appointment. While participating nurses were generally supportive of the study, it was evident that accommodating EPC within the context of their existing workload proved extremely challenging and ultimately unsustainable; this has the potential to undermine the viability of any future trial.

### Strengths and weaknesses

This external pilot study benefitted from a multi-methods approach, adopted to address clear and pre-specified research aims. We successfully developed and delivered an intervention providing EPC to patients who were depressed following an acute cardiac event, and who were attending a cardiac rehabilitation programme. Nested qualitative data found the intervention to be broadly acceptable to patients, and was welcomed by patients as reflecting a holistic approach to their care [23, 28, 29]. The intervention was developed by experts in the field and supported by the development of two manuals, targeting nurses and patients. Nurse-participants welcomed the opportunity of extending their clinical expertise, particularly in gaining confidence around the risk assessment of the psychological status of patients attending cardiac rehabilitation.

We successfully recruited cardiac rehabilitation teams and depressed participants being managed by these teams to the pilot study, although we were unable to meet recruitment targets, largely because of nurse-participant workload limiting nurses’ ability to accommodate the intervention within routine care, combined with a lower than anticipated rate of eligible and consenting patients.

In line with the original commissioning brief, our intention was to recruit patients who had evidence of new-onset depression occurring since the cardiac event, and as a result approximately a quarter of all patients identified with depressive symptoms during screening were excluded due to being actively treated for depression in the previous 6 months. While having an obvious impact on the sample size available for recruitment, it also possible that patients with pre-existing depression might also benefit from improved access to psychological care in cardiac rehabilitation settings, although this remains untested in our research.

Selection bias is a known feature of cluster randomised trials where participants are recruited after clusters have been randomised and the recruiting clinician is not blind to the treatment allocation [59]; to explore this, we compared the recruitment rates and participant characteristics at baseline between the trial arms. Recruitment rates were broadly comparable, although there was some imbalance in participant characteristics and outcomes at baseline between trial arms with EPC arm participants were younger, from more deprived areas, and with higher depression scores than UC participants. While these findings may have arisen by chance due to the small number of clusters and participants recruited into this pilot trial, the potential for selection bias remains an important design consideration for a future, definitive trial.

Although we reported effect sizes and associated 95% CIs for between-group differences in participant-reported outcomes (including the BDI-II), as this was a pilot study we did not use analytic methods that allow for clustering as the number of clusters and participants per cluster were too small. Our results provided important new data to inform future sample size calculations; however, we do not report *p* values for the observed effects and the confidence intervals must be interpreted with caution due to the lack of adjustment for clustering in the data.

We undertook follow-up of trial participants over a period of 8 months, and achieved good follow-up rates. Challenges of delayed recruitment, combined with funding constraints, meant that a small number of patients could only be followed up at our primary endpoint (5 months), rather than the full 8 months. Furthermore, we were unable to recruit patients from ethnic minority backgrounds, which potentially threatens the external validity of trial results, although the reasons why we were unable to do so were not entirely clear.

Substantial challenges were encountered in embedding supported EPC within routine cardiac rehabilitation, even when the intervention itself was largely patient-led, informed by principles of behaviour activation. Thus, while nurses and patients welcomed the focus on psychological aspects of care, workload challenges for cardiac rehabilitation nurses significantly limited the potential to effectively deliver this within the context of routine cardiac rehabilitation.

### Findings in context

In 2012, Leung et al. [6] reported the findings of a meta-analysis of 22 cohort studies investigating what timeframe around depression onset was associated with greater mortality and cardiac morbidity. Estimates of the prevalence of new-onset depression ranged from 5.5 to 27.9% and recurrent depression from 5.1 to 41.4%. The pooled analysis identified that both pre-ACS diagnosed depression and post-ACS diagnosed depression were potentially hazardous. Given this observation, excluding patients with pre-ACS depression from a future similar trial may be inappropriate.

The recently reported COBRA trial [64] identified the potential for effective psychological therapy targeting depression, such as we adopted here, to be delivered without the need for using costly and highly trained professionals. Given the key constraint of cardiac nurse workload identified in our study, embedding psychological practitioners within cardiac rehabilitation teams might better meet the mental health needs of such patients.

While the CADENCE study was ongoing, in 2016 Blumenthal et al. [65] published a RCT in the US that found that providing cardiac rehabilitation enhanced by stress management training delivered by psychological practitioners was associated with significant reductions in stress and the rate of adverse clinical events when compared with standard cardiac rehabilitation alone. Thus, although not directly addressing depression, Blumenthal and colleagues’ study provides further support for the incorporation of targeted psychological support for psychologically vulnerable groups of patients in the context of cardiac rehabilitation.

### Implications for future research

While EPC is desirable, embedding our nurse-supported EPC intervention within routine cardiac rehabilitation practice is not realistic in the current UK setting. One alternative might be use dedicated psychological-wellbeing practitioners, trained in the delivery of low-intensity psychological therapies, to deliver the intervention working closely with, and preferably embedded within, routine cardiac rehabilitation services. This change in EPC intervention would require new feasibility testing prior to undertaking a full-powered trial.

From a trial perspective, we successfully followed up patients at both 5 and 8 months. However, a future trial might consider a longer period of follow-up to allow sufficient time to accrue data on clinical events, although this would add considerably to the cost of a future trial. The BDI-II functioned effectively as an outcome for assessing the severity of depression, although consideration might be given to precisely which threshold might be implemented to secure a suitable group of patients for a future fully powered trial. Our data provide the basis for assessment of the size of a future fully powered study. It is likely that such a study would involve a cluster randomised design and include patients with pre-existing depression as well as those new-onset depression following a recent acute cardiac event. Given the observations reported here, it is likely that a future study would be considerably larger in scope; for example, potentially extending across a substantial proportion of cardiac rehabilitation teams currently operating in the UK.

## Conclusion

Cardiac rehabilitation nurses can be trained to deliver EPC. While valued by both patients and nurses, organisational and workload constraints are significant barriers to implementation. There remains a need to develop and test new models of psychological care within cardiac rehabilitation. This pilot study offers important data to inform the design and implementation of future trials of similar interventions to provide psychological care for patients with depression attending cardiac rehabilitation.

## Additional file

Additional file 1: **Table S1.** Session plan for nurses delivering enhanced psychological therapy (EPC) within a cardiac rehabilitation programme. **Table S2.** Outcome measures collected at each assessment. **Table S3.** Availability of biochemical and physiological outcome data. **Table S4.** Sample size calculations for a definitive cluster randomised trial. **Table S5.** Participant-reported health service use. **Table S6.** Comparison of data completeness between self-reported data and routine GP records: number of episodes/visits to different health services. (DOCX 67 kb)

## Abbreviations

ADM: Antidepressant medication
AE: Adverse event
BA: Behavioural activation
BACPR: British Association for Cardiovascular Prevention and Rehabilitation
BADS-SF: Behavioural Activation for Depression Scale – Short Form
BAI: Beck Anxiety Inventory
BDI-II: Beck Depression Inventory – Version 2
BMI: Body Mass Index
CABG: Coronary artery bypass grafting
CIS-R: Revised Clinical Interview Schedule
CSQ-8: Client Satisfaction Questionnaire-8 (CSQ-8)
CSRC: Clinically significant and reliable change
EQ-5D: EuroQoL Index
FFT: Friends and Family Test
IAPT: Improving Access to Psychological Therapies
ICC: Intra-cluster correlation coefficient
MCID: Minimal clinically important difference
MI: Myocardial infarction
NHS: National Health Service
NIHR: National Institute for Health Research
PCI: Percutaneous coronary intervention
PHQ-9: Patient Health Questionnaire-9
SAE: Serious adverse event
SD: Standard deviation
SRUQ: Service and Resource Use Questionnaire
UK: United Kingdom

## References

[1] NACR (2016). The National Audit of Cardiac Rehabilitation: Annual Statistical Report 2016.

[2] Connerney I, Shapiro PA, McLaughlin JS, Bagiella E, Sloan RP (2001). Relation between depression after coronary artery bypass surgery and 12-month outcome: a prospective study. Lancet..

[3] Rutledge T, Reis VA, Linke SE, Greenberg BH, Mills PJ (2006). Depression in heart failure a meta-analytic review of prevalence, intervention effects, and associations with clinical outcomes. J Am Coll Cardiol..

[4] Dickens C, Cherrington A, McGowan L (2012). Depression and health-related quality of life in people with coronary heart disease: a systematic review. Eur J Cardiovasc Nurs..

[5] Dickens CM, McGowan L, Percival C, Tomenson B, Cotter L, Heagerty A, Creed FH (2006). Contribution of depression and anxiety to impaired health-related quality of life following first myocardial infarction. Br J Psychiatry..

[6] Leung YW, Flora DB, Gravely S, Irvine J, Carney RM, Grace SL (2012). The impact of premorbid and postmorbid depression onset on mortality and cardiac morbidity among patients with coronary heart disease: meta-analysis. Psychosom Med..

[7] Dickens C, Katon W, Blakemore A, Khara A, McGowan L, Tomenson B, Jackson J, Walker L, Guthrie E (2012). Does depression predict the use of urgent and unscheduled care by people with long term conditions? A systematic review with meta-analysis. J Psychosom Res..

[8] Frasure-Smith N, Lesperance F, Gravel G, Masson A, Juneau M, Talajic M, Bourassa MG (2000). Depression and health-care costs during the first year following myocardial infarction. J Psychosom Res..

[9] Rutledge T, Vaccarino V, Johnson BD, Bittner V, Olson MB, Linke SE, Cornell CE, Eteiba W, Sheps DS, Francis J (2009). Depression and cardiovascular health care costs among women with suspected myocardial ischemia: prospective results from the WISE (Women’s Ischemia Syndrome Evaluation) Study. J Am Coll Cardiol..

[10] Barth J, Schumacher M, Herrmann-Lingen C (2004). Depression as a risk factor for mortality in patients with coronary heart disease: a meta-analysis. Psychosom Med..

[11] Gale CR, Batty GD, Osborn DP, Tynelius P, Rasmussen F (2014). Mental disorders across the adult life course and future coronary heart disease: evidence for general susceptibility. Circulation..

[12] Meijer A, Conradi HJ, Bos EH, Thombs BD, van Melle JP, de Jonge P (2011). Prognostic association of depression following myocardial infarction with mortality and cardiovascular events: a meta-analysis of 25 years of research. Gen Hosp Psychiatry..

[13] Nicholson A, Kuper H, Hemingway H (2006). Depression as an aetiologic and prognostic factor in coronary heart disease: a meta-analysis of 6362 events among 146,538 participants in 54 observational studies. Eur Heart J..

[14] van Melle JP, de Jonge P, Spijkerman TA, Tijssen JG, Ormel J, van Veldhuisen DJ, van den Brink RH, van den Berg MP (2004). Prognostic association of depression following myocardial infarction with mortality and cardiovascular events: a meta-analysis. Psychosom Med..

[15] British Association for Cardiovascular Prevention and Rehabilitation. The BACPR Standards and Core Components for Cardiovascular Disease Prevention and Rehabilitation 2017. London: British Cardiovascular Society; 2017.

[16] Pogosova N, Saner H, Pedersen SS, Cupples ME, McGee H, Hofer S, Doyle F, Schmid JP, von Kanel R (2015). Cardiac Rehabilitation Section of the European Association of Cardiovascular P, et al. Psychosocial aspects in cardiac rehabilitation: from theory to practice. A position paper from the Cardiac Rehabilitation Section of the European Association of Cardiovascular Prevention and Rehabilitation of the European Society of Cardiology. Eur J Prev Cardiol..

[17] Piepoli MF, Corra U, Adamopoulos S, Benzer W, Bjarnason-Wehrens B, Cupples M, Dendale P, Doherty P, Gaita D, Hofer S (2014). Secondary prevention in the clinical management of patients with cardiovascular diseases. Core components, standards and outcome measures for referral and delivery: a policy statement from the cardiac rehabilitation section of the European Association for Cardiovascular Prevention and Rehabilitation. Endorsed by the Committee for Practice Guidelines of the European Society of Cardiology. Eur J Prev Cardiol.

[18] Lichtman JH, Froelicher ES, Blumenthal JA, Carney RM, Doering LV, Frasure-Smith N, Freedland KE, Jaffe AS, Leifheit-Limson EC, Sheps DS (2014). Depression as a risk factor for poor prognosis among patients with acute coronary syndrome: systematic review and recommendations: a scientific statement from the American Heart Association. Circulation..

[19] National Institute for Clinical Excellence Commissioning Guidance. Myocardial infarction: cardiac rehabilitation and prevention of further cardiovascular disease. London: NICE; 2013. 31891465

[20] Dickens C (2015). Depression in people with coronary heart disease: prognostic significance and mechanisms. Curr Cardiol Rep..

[21] Richards SH, Anderson L, Jenkinson CE, Whalley B, Rees K, Davies P, Bennett P, Liu Z, West R, Thompson DR et al. Psychological interventions for coronary heart disease. Cochrane Database Syst Rev. 2017; 4(Issue 4. Art. No.: CD002902):CD002902. 10.1002/14651858.CD002902.pub4 PMC6478177 28452408

[22] Richards S, Dickens C, Anderson R, Richards D, Taylor R, Ukoumunne O, Kessler D, Turner K, Kuyken W, Gandhi M (2016). Assessing the effectiveness of enhanced psychological care for patients with depressive symptoms attending cardiac rehabilitation compared with treatment as usual (CADENCE): study protocol for a pilot cluster randomised controlled trial. Trials..

[23] Turner KM, Winder R, Campbell JL, Richards DA, Gandhi M, Dickens CM, Richards S (2017). Patients’ and nurses’ views on providing psychological support within cardiac rehabilitation programmes: a qualitative study. BMJ Open..

[24] Thabane L, Ma J, Chu R, Cheng J, Ismaila A, Rios L, Robson R, Thabane M, Giangregorio L, Goldsmith C (2010). A tutorial on pilot studies: the what, why and how. BMC Med Res Methodol..

[25] Lancaster GA, Dodd S, Williamson PR (2004). Design and analysis of pilot studies: recommendations for good practice. J Eval Clin Pract..

[26] British Association for Cardiovascular Prevention and Rehabilitation. The BACPR Standards and Core Components for Cardiovascular Disease Prevention and Rehabilitation 2012. 2nd ed. London: British Cardiovascular Society; 2012.

[27] NACR. National Audit of Cardiac Rehabilitation: 2012. British Heart Foundation, London, UK; 2012.

[28] Winder R, Richards SH, Campbell JL, Richards DA, Dickens C, Gandhi M, Wright C, Turner K (2017). Development and refinement of a complex intervention within cardiac rehabilitation services: experiences from the CADENCE feasibility study. Pilot Feasibil Stud..

[29] Richards S, Campbell J, Dickens C, Anderson R, Kessler D, Gandhi M, Gibson A, Knight L, Kuyken W, Richards D, et al. A feasibility study and pilot RCT to establish methods for assessing the acceptability, and clinical effectiveness and cost effectiveness of enhanced psychological care in cardiac rehabilitation for patients with new onset depressive symptoms compared with treatment as usual: the CADENCE study. Health Technol Assess. 2018. In press. 10.3310/hta22300 PMC6004545 29856312

[30] National Collaborating Centre for Mental Health (2009). Depression in adults with a chronic physical health problem (NICE Clinical Guideline 91).

[31] National Collaborating Centre for Mental Health (2009). Depression: The treatment and management of depression in adults (NICE Clinical Guideline 90).

[32] Addis M, Martell C. Overcoming depression one step at a time: the new behavioral activation approach to getting your life back. Oakland: New Harbinger Publications; 2004.

[33] Cuijpers P, van Straten A, Warmerdam L (2007). Behavioral activation treatments of depression: a meta-analysis. Clin Psychol Rev..

[34] Richards D, Bennett-Levy J, Richards D, Farrand P, Christensen H, Griffiths K, Kavanagh D, Klein B, Proudfoot J, White J, Williams C (2010). Behavioural Activation. Oxford guide to low intensity CBT interventions.

[35] Richards D, Farrand P, Chellingsworth M (2011). National Curriculum for the Education of Psychological Wellbeing Practitioners (PWPS).

[36] Ekers D, Richards D, Gilbody S (2008). A meta-analysis of randomized trials of behavioural treatment of depression. Psychol Med..

[37] Ekers D, Richards D, McMillan D, Bland JM, Gilbody S (2011). Behavioural activation delivered by the non-specialist: phase II randomised controlled trial. Br J Psychiatry..

[38] Lowe B, Unutzer J, Callahan CM, Perkins AJ, Kroenke K (2004). Monitoring depression treatment outcomes with the Patient Health Questionnaire-9. Med Care..

[39] Kroenke K, Spitzer RL, Williams JB (2001). The PHQ-9: validity of a brief depression severity measure. J Gen Intern Med..

[40] Meader N, Mitchell AJ, Chew-Graham C, Goldberg D, Rizzo M, Bird V, Kessler D, Packham J, Haddad M, Pilling S (2011). Case identification of depression in patients with chronic physical health problems: a diagnostic accuracy meta-analysis of 113 studies. Br J General Pract..

[41] Zigmond AS, Snaith RP (1983). The Hospital Anxiety and Depression Scale. Acta Psychiatr Scand..

[42] Department for Communities and Local Government. The English Indices of Deprivation 2010. London: Department for Communities and Local Government; 2010. p. 1–20.

[43] Lewis G (1994). Assessing psychiatric disorder with a human interviewer or a computer. J Epidemiol Community Health..

[44] Lewis G, Pelosi AJ, Araya R, Dunn G (1992). Measuring psychiatric disorder in the community: a standardized assessment for use by lay interviewers. Psychol Med..

[45] King M, Nazareth I, Lampe F, Bower P, Chandler M, Morou M, Sibbald B, Lai R (2005). Conceptual framework and systematic review of the effects of participants’ and professionals’ preferences in randomised controlled trials. Health Technol Assess.

[46] Morisky DE, Green LW, Levine DM (1986). Concurrent and predictive validity of a self-reported measure of medication adherence. Med Care..

[47] Beck AT, Steer RA, Brown GK (1996). Beck Depression Inventory: manual.

[48] Beck CA, Joseph L, Belisle P, Pilote L, Investigators Q (2001). Predictors of quality of life 6 months and 1 year after acute myocardial infarction. Am Heart J..

[49] Beck AT, Steer RA (1990). Manual for the Beck Anxiety Inventory.

[50] Beck AT, Steer RA, Ball R, Ciervo CA, Kabat M (1997). Use of the Beck Anxiety and Depression Inventories for primary care with medical outpatients. Assessment..

[51] The EuroQol Group (1996). EQ-5D user guide.

[52] Devlin N, Shah K, Feng Y, Mulhern B, Van Hout B (2016). Valuing health-related quality of life: an EQ-5D-5L value set for England.

[53] Oldridge N, Hofer S, McGee H, Conroy R, Doyle F, Saner H (2014). The HeartQoL: part II. Validation of a new core health-related quality of life questionnaire for patients with ischemic heart disease. Eur J Prev Cardiol..

[54] Oldridge N, Hofer S, McGee H, Conroy R, Doyle F, Saner H (2014). The HeartQoL: Part I. Development of a new core health-related quality of life questionnaire for patients with ischemic heart disease. Eur J Prev Cardiol..

[55] Manos RC, Kanter JW, Luo W (2011). The behavioral activation for depression scale-short form: development and validation. Behav Ther..

[56] Attkisson CC, Zwick R (1982). The Client Satisfaction Questionnaire. Psychometric properties and correlations with service utilization and psychotherapy outcome. Eval Prog Plann..

[57] Beecham J, Knapp M (1992). Costing psychiatric interventions. Measuring mental health needs.

[58] Campbell MK, Piaggio G, Elbourne DR, Altman DG, Group C (2012). Consort 2010 statement: extension to cluster randomised trials. BMJ..

[59] Eldridge S, Kerry S, Torgerson DJ (2009). Bias in identifying and recruiting participants in cluster randomised trials: what can be done?. BMJ..

[60] Bockting CL, Hollon SD, Jarrett RB, Kuyken W, Dobson K (2015). A lifetime approach to major depressive disorder: the contributions of psychological interventions in preventing relapse and recurrence. Clin Psychol Rev..

[61] Button KS, Kounali D, Thomas L, Wiles NJ, Peters TJ, Welton NJ, Ades AE, Lewis G (2015). Minimal clinically important difference on the Beck Depression Inventory—II according to the patient’s perspective. Psychol Med..

[62] Jacobson NS, Truax P (1991). Clinical significance: a statistical approach to defining meaningful change in psychotherapy research. J Consult Clin Psychol..

[63] Gibson A, Britten N, Lynch J (2012). Theoretical directions for an emancipatory concept of patient and public involvement. Health (London)..

[64] Richards DA, Ekers D, McMillan D, Taylor RS, Byford S, Warren FC, Barrett B, Farrand PA, Gilbody S, Kuyken W (2016). Cost and Outcome of Behavioural Activation versus Cognitive Behavioural Therapy for Depression (COBRA): a randomised, controlled, non-inferiority trial. Lancet..

[65] Blumenthal JA, Sherwood A, Smith PJ, Watkins L, Mabe S, Kraus WE, Ingle K, Miller P, Hinderliter A (2016). Enhancing cardiac rehabilitation with stress management training: a randomized, clinical efficacy trial. Circulation..

