# Supplementary material for: Assessing the effectiveness of Enhanced Psychological Care for patients with depressive symptoms attending cardiac rehabilitation compared with treatment as usual (CADENCE): a pilot cluster randomised controlled trial
Source: Trials. 2018 Apr 2;19:211. doi: 10.1186/s13063-018-2576-9 (PMC5880097; doi:10.1186/s13063-018-2576-9)
Supplement: Supplementary file 1 — Table S1. Session plan for nurses delivering enhanced psychological therapy (EPC) within a cardiac rehabilitation programme. Table S2. Outcome measures collected at each assessment. Table S3. Availability of biochemical and physiological outcome data. Table S4. Sample size calculations for a definitive cluster randomised trial. Table S5. Participant-reported health service use. Table S6. Comparison of data completeness between self-reported data and routine GP records: number of episodes/visits to different health services. (DOCX 67 kb) [file 13063_2018_2576_MOESM1_ESM.docx]

**Additional file 1**

**Table S1. Session plan for nurses delivering EPC within a cardiac rehabilitation programme**

| **First cardiac rehabilitation session:** Explain to participant the options available i.e. supported self-help BA manual, with or without onward referral to relevant mental health care services, depending on individual preferences. Agree mental health treatment plan and take relevant action. This might include: |
| --- |
| - agreeing to discuss the self-help BA book next time you meet (or arranging a special follow up telephone appointment) |
| - writing to or telephoning the patient’s GP |
| - giving out details of the local IAPT service or making a referral yourself |
| - arranging a specialist cardiac psychological therapy referral |
| **All remaining sessions:** Content tailored depending on whether the participant has decided to follow self-help BA manual. These sessions can be brief depending on patient progress. |
| *Care co-ordination only*. Review mood since last appointment, using PHQ-9 and GAD-7 if preferred. Check that the patient’s mood is not deteriorating further, that they are safe and to see if the action you agreed with the patient to take has been followed up (i.e. did the participant make the GP appointment?). |
| *Self-help BA manual.* At each session assess symptoms and risk, review treatment choices, support BA, and future planning. BA support is aimed at helping participants to engage with the self-help manual, explaining ideas and methods as required. |
| At **the mid point (around 4 weeks)** dedicated clinic time should be allocated to reviewing progress and carefully review treatment options. |
| At the **final session (around week 6-8)** dedicated time should be allocated to reviewing progress, and structured details of the care received will be sent to their GP. Participants failing to respond to self-help BA will be referred on to their preferred management option. |

**Table S2. Outcome measures collected at each assessment**

| **Measure** | **Baseline** | **Five months** | **Eight months** |
| --- | --- | --- | --- |
| CIS-R | ✓ | - | - |
| BDI | ✓ | ✓ | ✓ |
| BAI | ✓ | ✓ | ✓ |
| EQ-5D | ✓ | ✓ | ✓ |
| HeartQoL | ✓ | ✓ | ✓ |
| CSQ | - | ✓ | - |
| Friends and Family | - | ✓ | - |
| Service Resource Use Questionnaire | - | ✓ | ✓ |
| Cardiac nurse case notes review | - | **-** | ✓ |
| GP case notes review | - | **-** | ✓ |

**Table S3. Availability of biochemical and physiological outcome data**

|  | **Baseline^a^** | | **Five months^b^** | | **Eight months^c^** | |
| --- | --- | --- | --- | --- | --- | --- |
| **Outcome measure availability^d^; n (%)** | UC  N=14 | EPC  N=15 | UC  N=14 | EPC  N=15 | UC  N=8 | EPC  N=9 |
| BMI | 9 (64) | 6 (40) | 9 (64) | 5 (33) | 1 (13) | 4 (44) |
| Blood Pressure | 13 (93) | 13 (87) | 13 (93) | 15 (100) | 4 (50) | 7 (78) |
| HbA1c | 1 (7) | 2 (13) | 4 (29) | 1 (7) | 0 (0) | 1 (11) |
| Total cholesterol | 7 (50) | 8 (53) | 9 (64) | 6 (40) | 2 (14) | 0 (0) |
| Triglycerides | 2 (14) | 2 (13) | 5 (36) | 1 (7) | 1 (13) | 1 (11) |

^a^ Most recent measurement between indicating cardiac event and baseline.

^b^ Most recent measurement between baseline and five months.

^c^ Most recent measurement between five months and eight months.

^d^ Data available in GP notes and/or cardiac rehabilitation team notes (data source not reported).

**Table S4. Sample size calculations for a definitive cluster randomised trial**

| **Scenario** | **BDI-II score** | | **Clinically meaningful effect (MCID)**^a^ | | **Required total  sample size** | |
| --- | --- | --- | --- | --- | --- | --- |
|  | **Mean** | **SD** | **BDI-II scale** | **Effect size** | **Cardiac teams** | **Participants** |
| 1 | 6 | 3 | 1.05 | 0.35 | 54 | 702 |
| 2 | 6 | 3.5 | 1.05 | 0.3 | 72 | 936 |
| 3 | 6 | 5 | 1.05 | 0.21 | 148 | 1924 |
| 4 | 7 | 3 | 1.225 | 0.41 | 40 | 520 |
| 5 | 7 | 3.5 | 1.225 | 0.35 | 54 | 702 |
| 6 | 7 | 5 | 1.225 | 0.25 | 104 | 1352 |
| 7 | 8 | 3 | 1.4 | 0.47 | 30 | 390 |
| 8 | 8 | 3.5 | 1.4 | 0.4 | 42 | 546 |
| 9 | 8 | 5 | 1.4 | 0.28 | 84 | 1092 |

^a^ Estimates are based on different assumed true values of the mean and standard deviation of the BDI-II measure for the control group at eight months. We assumed an intra-cluster correlation coefficient (ICC) of 0.047 based on national audit data.

**Table S5. Participant reported health service use**

| **Time period** | **UC, N=14** | | **EPC, N=13** | |
| --- | --- | --- | --- | --- |
| **From baseline to five months** | **Participants with >1 contact, n (%)** | **Mean cost £ (SD)** | **Participants with >1 contact, n (%)** | **Mean cost £ (SD)** |
| GP visits (incl. practice nurses) | 14 (100) | 335 (276) | 13 (100) | 445 (307) |
| Seeing other care professionals | 5 (38) | 31 (62) | 8 (57) | 550 (1542) |
| Hospital inpatients | 4 (29) | 456 (784) | 4 (31) | 1246 (1879) |
| Hospital outpatients | 10 (71) | 170 (213) | 11 (85) | 194 (161) |
| Emergency care (eg. ED, 999) | 3 (21) | 28 (54) | 8 (62) | 82 (110) |
| **Mean total cost of health service use** |  | **1020 (955)** |  | **2516 (3044)** |
|  | **UC, N=8** | | **EPC, N=9** | |
| **From five months to eight months** | **Participants with >1 contact, n (%)** | **Mean cost £ (SD)** | **Participants with *>*1 contact, n (%)** | **Mean cost £ (SD)** |
| GP visits (incl. practice nurses) | 8 (100) | 211 (123) | 9 (100) | 170 (105) |
| Seeing other care professionals | 1 (13) | 6 (16) | 3 (33) | 56 (95) |
| Hospital inpatients | 1 (13) | 221 (585) | 0 | 0 |
| Hospital outpatients | 4 (50) | 157 (207) | 5 (56) | 130 (175) |
| Emergency care (eg. Emergency department visit) | 2 (25) | 70 (124) | 1 (11) | 12 (35) |
| **Mean total cost of health service use** |  | 664 (822) |  | 368 (265) |

**Table S6. Comparison of data completeness between self-reported data and routine GP records: number of episodes/visits to different health services**

| **Professional seen or service visited** | **Mean (SD) no. visits: Self-report** | **Mean (SD) no. visits: GP records** | **ICC^a^** | **Crude agreement; n (%)** | **Participant underestimate^b^;**  **n (%)** | **Participant overestimate^b^;**  **n (%)** |
| --- | --- | --- | --- | --- | --- | --- |
| GP (surgery visits) | 6.1 (5.7) | 6.1 (3.8) | 0.34 | 0 (0) | 9 (53) | 8 (47) |
| Practice nurse visits | 4.7 (5.4) | 3.9 (5.1) | 0.62 | 3 (18) | 7 (41) | 7 (41) |
| Emergency department visits | 0.6 (1.1) | 0.7 (1.5) | 0.70 | 11 (65) | 4 (24) | 2 (12) |
| Hospital admissions | 0.5 (0.9) | 0.4 (1.0) | 0.85 | 14 (82) | 1 (6) | 2 (12) |

^a^ ICC=Intraclass correlation coefficient

^b^ Participant under- or over-estimating number of events when compared with GP records
